# Analysis of antimicrobial efficacy of sodium hypochlorite and ozonated water against biofilm in oval canals

**DOI:** 10.1590/0103-6440202305318

**Published:** 2023-07-17

**Authors:** Ivy Rodrigues Merçon, Francisco Ubiratan Ferreira de Campos, Carlos Eduardo Fontana, Rina Andréa Pelegrine, Alexandre Sigrist De Martin, Carlos Eduardo da Silveira Bueno

**Affiliations:** 1 Faculdade São Leopoldo Mandic, Instituto de Pesquisa São Leopoldo Mandic., Campinas, SP, Brasil.; 2 Graduate Program in Health Sciences, Pontifícia Universidade Católica de Campinas(PUC Campinas), Campinas, SP, Brasil.

**Keywords:** biofilm, apical periodontitis, disinfection, sodium hypochlorite, ozone

## Abstract

This *in vitro* study compared the antimicrobial efficacy of 2.5% sodium hypochlorite (NaOCl) and 8 µg/mL ozonated water agitated by passive ultrasonic irrigation (PUI) or PUI combined with EndoActivator (EA) against mature multispecies biofilm. One hundred and five oval-shaped mandibular premolars were instrumented, sterilized, and inoculated with *Enterococcus faecalis, Candida albicans*, and *Staphylococcus aureus,* divided into: control group - saline; O_3_ group - ozonated water; O_3_ PUI group - ozonated water with PUI agitation; O_3_ PUI+EA group - ozonated water with PUI+EA agitation; NaOCl group - NaOCl; NaOCl PUI group - NaOCl with PUI agitation; and NaOCl PUI+EA group - NaOCl with PUI+EA agitation. Microbiological samples were collected before (S1) and after (S2) the disinfection procedures and the data were statistically analyzed using the Kruskal-Wallis test. In the culture method, there was significant disinfection in the O_3_ PUI+EA, NaOCl, NaOCl PUI, and NaOCl PUI+EA groups (p˂0.05). The combination of NaOCl with PUI+EA reduced microbial counts to zero (p˂0.05). In the qPCR method, there was a significant reduction in the total count of viable microorganisms in the O_3_ PUI, O_3_ PUI+EA, NaOCl, NaOCl PUI, and NaOCl PUI+EA groups (p˂0.05). It can be concluded that 2.5% NaOCl with and without agitation, as well as 8 µg/mL ozonated water with its action enhanced by the agitation techniques, were effective in root canal disinfection, and their antimicrobial efficacy is related to the microorganisms present in the biofilm.

## Resumo

Este estudo *in vitro* comparou a desinfecção do hipoclorito de sódio 2,5% (NaOCl) e da água ozonizada 8 µg/mL agitados pela irrigação ultrassônica passiva (PUI) e por associação da PUI com EndoActivator (EA) na redução de biofilme misto maduro. Cento e cinco pré-molares inferiores ovalados foram instrumentados, esterilizados e inoculados com Enterococcus faecalis, Candida albicans e Staphylococcus aureus, divididos em: Grupo controle: soro; Grupo O3: água ozonizada; Grupo O3 PUI: água ozonizada agitada por PUI; Grupo O3 PUI + EA: água ozonizada agitada por PUI e EA: Grupo NaOCl: hipoclorito de sódio; Grupo NaOCl PUI: hipoclorito de sódio agitado por PUI; Grupo NaOCl PUI + EA: hipoclorito de sódio agitado por PUI e EA. Amostras microbiológicas foram coletadas antes (S1) e após (S2) os procedimentos de desinfecção e os dados foram analisados estatisticamente pelo teste de Kruskal-Wallis. No método de cultura, houve desinfecção significativa nos grupos O3 PUI + EA, NaOCl, NaOCl PUI e NaOCl PUI + EA (p˂0.05), sendo que no grupo NaOCl PUI + EA não houve crescimento de microrganismo (p˂0.05). No método de qPCR, nas contagens dos microrganismos antes e após os protocolos de desinfecção, houve redução microbiana nos grupos O3 PUI, O3 PUI + EA, NaOCl, NaOCl PUI, NaOCl PUI + EA (p˂0.05). Concluiu-se que o NaOCl 2,5% com e sem agitação foi eficiente, assim como a água ozonizada 8 µg/mL potencializada pelos métodos de agitação na desinfecção e que a mesma está relacionada com os microrganismos presentes no biofilme.

## Introduction

Apical periodontitis is an infectious disease caused by microorganisms that colonize the root canal system. To improve the prognosis of endodontic treatment, bacterial populations should ideally be eliminated or reduced to biologically compatible levels to permit the periapical tissues to heal. Bacterial persistence after chemomechanical preparation, supplemented or not with intracanal medicament, poses an increased risk of adverse prognosis in endodontic treatment. Furthermore, the presence of bacteria in the root canal at the time of filling has been shown to be a risk factor for post-treatment apical periodontitis ^1^.

Approximately 150 species of microorganisms can colonize the root canal system, and some of them can induce or maintain periapical lesions ^2^. *Enterococcus faecalis* is a Gram-positive bacterium that appears individually and usually occurs in pairs or short chains. This facultative anaerobic bacterium is commonly isolated from root canals with persistent periapical disease. *E. faecalis* can colonize dentin, invade dentinal tubules, and resist the antimicrobial actions of irrigating solutions and intracanal medicaments ^3^. *Candida albicans* is a eukaryotic fungus found in infected root canals, with a prevalence ranging from 0.5% to 5.5%, mainly in secondary/persistent infections, and virulence factors that may play an important role in the onset of endodontic pathologies ^4^. *Staphylococcus aureus* is a Gram-positive, facultative bacterium that produces enzymes potentially important in microbial pathogenicity, such as coagulase, hyaluronidase, lysozyme, and lipases, as well as several toxins, such as hemolysins, epidermolytic toxin, and some enterotoxins, causing several diseases ^5^. To reproduce a polymicrobial biofilm, which is what occurs in endodontic infections, *E. faecalis, C. albicans,* and *S. aureus* were selected because they represent species that are resistant to disinfection protocols, often being present in secondary/persistent infections ^1^.

In endodontics, irrigation is an integral part of canal preparation, playing a critical role in disinfection and debris removal. Sodium hypochlorite (NaOCl) has been widely used as an irrigant in endodontic treatment due to its ability to dissolve tissue and excellent antimicrobial activity. However, a major drawback is its toxicity to periapical tissues when it extrudes through the apex ^6^. Ozone therapy is a technique that uses a mixture of oxygen and ozone (O_3_), a naturally occurring gas and a strong and selective oxidant, for therapeutic purposes ^7^. It is based on the assumption that O_3_ rapidly dissociates into water and releases a reactive form of oxygen that can oxidize cells, thus exhibiting antimicrobial activity without inducing drug resistance ^8^. However, the main disadvantage is long-term concentration instability ^6^. Due to the widespread use of NaOCl, despite its cytotoxicity, the study of new irrigating agents is relevant. Ozonated water has important characteristics such as disinfection potential and biocompatibility, but it needs further research.

Not only the irrigant but also the irrigation technique plays an important role. Conventional syringe-needle irrigation cannot clean hard-to-reach areas of the canal, being considered insufficient for complete cleaning of the root canal space ^6^. Several techniques and devices have been proposed to improve irrigation efficiency, including sonic or ultrasonic agitation ^9^.

Passive ultrasonic irrigation (PUI) is the most commonly used technique for agitation of the chemical irrigant. PUI promotes a cavitation effect by producing bubbles that burst close to the dentin walls, in addition to generating microacoustic streaming that promotes the hydrodynamic agitation of the irrigant, enhancing cleaning, but it has the limitation of the ultrasonic insert being positioned 2 mm short of the working length in straight canals and up to the beginning of the curvature in curved canals ^10^. The EndoActivator (Dentsply, Tulsa Dental Specialties, Tulsa, USA) is a device composed of a sonic energy-generating handpiece, which operates at low frequency and high amplitude, and flexible medical grade polymer tips, which can be used within the working length. Sonic agitation does not create cavitation or microacoustic streaming but rather promotes irrigant penetration into the dentinal tubules and removal of debris and smear layer, in addition to the possibility of agitating the irrigant in the working length of straight and curved canals ^11^. As exposed, both techniques have limitations and, to the best of our knowledge, the hybridization of these methods is something not found in the literature, which is an innovative aspect of the present study.

In view of the foregoing, the eradication of multispecies biofilms containing resistant microorganisms is crucial for successful endodontic treatment. Therefore, the purpose of this study was to compare the antimicrobial efficacy of 2.5% NaOCl and 8 µg/mL ozonated water agitated by PUI or PUI combined with sonic agitation using the EndoActivator (EA) system against mature multispecies biofilms containing *E. faecalis, C. albicans*, and *S. aureus*. The null hypotheses tested for each method, culture and molecular (quantitative polymerase chain reaction [qPCR]), were that ^1^ both irrigants (NaOCl and ozonated water) would have equivalent root canal disinfection efficiency and ^2^ the irrigation protocols tested (conventional syringe-needle irrigation, PUI, and PUI+EA) would be equivalent in their ability to enhance irrigant action.

## Materials and methods

The local research ethics committee (protocol number 5.129.512) approved this study. The sample size of 15 specimens per group was based on the results of a pilot test and the study by Moraes et al. ^12^. The sample size was calculated by analysis of variance (ANOVA) for a minimum difference between the treatment means of 0.12, an error deviation of 0.085, number of treatments of 7, power of 0.80, and alpha of 0.05.

The teeth were obtained from the Biorepository Bank of the São Leopoldo Mandic Dental Research Center, Campinas, SP, Brazil. Mandibular premolars extracted for orthodontic, prosthetic, or periodontal indication were selected after obtaining written informed consent from the patient. Inclusion criteria were single-rooted teeth with a fully formed apex and curvature angle of 0º to 5º, according to Schneider ^13^. For this assessment, the teeth were radiographed both in the buccolingual and mesiodistal directions, and a line was drawn parallel to the long axis of the canal. A second line was drawn from the apical foramen to intersect the first line at the point where the canal began to leave the long axis of the tooth. The acute angle formed was measured using a protractor. According to the degree of curvature, the roots were classified as straight (5º or less), moderately curved (10º to 20º), or severely curved (25º to 70º). A single oval-shaped canal and no internal root resorptions or calcifications were also considered based on the analysis of the radiographs taken in buccolingual and mesiodistal directions. The oval shape was characterized when the buccolingual diameter was twice as large as the mesiodistal diameter at the cervical third of the root canal. All specimens were stored in the saline solution until use to prevent dehydration and were examined under an operating microscope (Alliance Microscopia e Colposcopia, São Carlos, SP, Brazil) at 8× magnification to exclude those with fractures or cracks.

### Sample preparation

A total of 105 single roots from extracted mandibular premolars were used. The sample was prepared by a single experienced endodontist. The crowns were sectioned using Zekrya burs (Dentsply, Maillefer, Ballaigues, Switzerland) driven by a high-speed motor (Dabi Atlante, Ribeirão Preto, SP, Brazil), under water cooling. The tooth length was standardized at 15 mm.

The root canals were initially prepared with a #10 K-file (Dentsply, Maillefer) with oscillatory movements until 2 mm short of the initial tooth length, followed by a #15 K-file (Dentsply, Maillefer) until 5 mm short of the initial tooth length, also with oscillatory movements. The canals were instrumented with the Protaper Next rotary system (Dentsply, Maillefer) driven by the X-Smart Plus motor (Dentsply, Maillefer) at a speed of 300 rpm and a torque of 2 Ncm in an in-and-out pecking motion, combined with a brushing motion. Each file was used 4 times and then discarded. Pre-flaring was performed with the X1 file (17.04) until 5 mm short of the initial tooth length. A #10 K-file was inserted into the root canal until its tip was visible at the apical foramen, and the working length was visually determined at 1 mm short of the foramen. The anatomic diameter of the root canal corresponded to a #15 K-file (Dentsply, Maillefer). The canal was prepared with a #15 K-file to the working length and then instrumented with X1 (17.04), X2 (25.06), and X3 (30.07) files also to the working length. At each instrument change, a #10 K-file was inserted to a length of 1 mm beyond the apical foramen to ensure patency, and the specimens were irrigated with 3 mL of 2.5% NaOCl (Fórmula & Ação, São Paulo, SP, Brazil), for a total of 25 mL, using a disposable syringe and 0.55 x 20 mm needle (Embramac Empresa Brasileira de Material Cirúrgico Indústria e Comércio Importação e Exportação, Campinas, SP, Brazil). The flutes of the instrument were cleaned with gauze.

The smear layer was removed by irrigating the canals with 3 mL of 17% EDTA for 3 minutes, followed by aspirtion with a 0.014ʺ capillary tip (Ultradent do Brasil, Indaiatuba, SP, Brazil). Finally, the canals were irrigated with 3 mL of 2.5% NaOCl, also for 3 minutes, and aspirated with a 0.014ʺ capillary tip. All canals were dried with sterile absorbent paper points (Tanari, Manacapuru, AM, Brazil).

The apical foramen of all teeth was sealed with light-cured composite resin (Filtek Z250; 3M ESPE, St Paul, MN, USA) to prevent bacteria from entering through the foramen and to create a closed system. Two layers of nail polish were applied to the outer surface of all roots. Heavy-body silicone putty (Speedex, Coltene; Vigodent S/A, Rio de Janeiro, RJ, Brazil) was used to create niches for the roots, which were inserted into 24-well cell culture plates for preparation of test specimens and bacterial colonization. The specimens were autoclaved at 121°C for 15 minutes and then incubated at 37°C for 24 hours to evaluate the occurrence of bacterial growth.

### Specimen contamination

Standard strains of *E. faecalis* (ATCC 29212), *S. aureus* (ATCC 25923), and *C. albicans* (ATCC 10231) were activated in brain-heart infusion (BHI) broth (Difco, Detroit, MI, USA), matched to a 10 McFarland standard - suspension containing 3.0 × 10^9^ colony forming units (CFUs) per mL. The colonies were injected into the root canals using a sterile insulin syringe with a 30-gauge needle and incubated at 37°C for 21 days. During this period, a 20-µL aliquot of the suspension was replaced daily for each specimen using a sterile pipette in a laminar flow hood under a 5% CO_2_ atmosphere. Two random specimens were checked weekly for the viability of microorganisms by inserting a sterile paper point into the root canal and then incubating it at 37°C for 24 hours.

### Preparation of ozonated water

Ozonated water was prepared following the protocol described by Nogales et al. ^14^ using an ozone generator with a self-calibration system at a flow rate of 1 L/min. Ozone was generated by passing an electrical discharge through medical oxygen molecules. A 50-cm-high glass column was connected to the generator (Philozon, Balneário Camboriú, SC, Brazil), and ozone was bubbled into reverse osmosis water cooled at 14°C for 5 minutes. The generator was calibrated to produce 40 µg/mL of ozone, with a final aqueous ozone concentration of 8 µg/mL, which was used immediately after preparation.

### Experimental groups and treatments

Microbiological samples were collected, under aseptic conditions in a laminar flow hood under a 5% CO_2_ atmosphere, before (S1) and after (S2) the study disinfection procedures. The first sample (S1) was collected by inserting a sterile FM paper point (Tanari) to the working length for 1 minute and then placing it in an Eppendorf tube containing 900 µL of saline solution, which was vortex mixed for 30 seconds. The bacterial suspension was serially diluted at 10^-1^, 10^-2^, 10^-3^, and 10^-4^ concentrations, and aliquots were seeded into Petri dishes containing BHI agar and incubated at 37ºC under a 5% CO_2_ atmosphere for 24 hours. Microbial growth was measured by the CFU/mL counts. CFU counting was performed by a single calibrated and trained operator using a digital colony counter (Cp-600 Plus Tecnal, Piracicaba, SP, Brazil).

**Figure 1:**
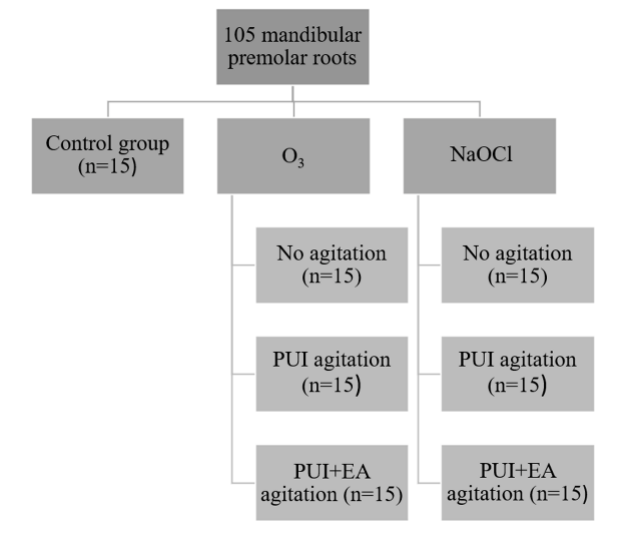
Flowchart of the study disinfection protocols

The roots were randomly divided into 7 groups (n=15 each) according to the treatment performed, as shown in Figure 1. All disinfection procedures were also performed under aseptic conditions in a laminar flow hood under a 5% CO_2_ atmosphere.

-Negative control group (saline) - the canals were irrigated with 5 mL of sterile saline solution for 1 minute, using a 0.55 x 20 mm needle (Embramac) inserted to a length of 10 mm.

-NaOCl group - the canals were irrigated with 5 mL of 2.5% NaOCl (Fórmula & Ação) for 1 minute, using a 0.55 x 20 mm needle (Embramac) inserted to a length of 10 mm.

-NaOCl PUI group - the canals were irrigated with 1.6 mL of 2.5% NaOCl (Fórmula & Ação), using a 0.55 x 20 mm needle (Embramac) inserted to a length of 10 mm, activated using an Irrisonic tip (Helse Ultrasonic, Santa Rosa de Viterbo, São Paulo, SP, Brazil) coupled to an ultrasonic piezoelectric unit (Azultrasonic, J. Morita, California, USA) at 30% power, inserted into the root canal until 2 mm short of the working length for 20 seconds and aspirated with a needle and syringe (Embramac). NaOCl was replaced with fresh irrigant and agitation was repeated 3 times, for 20 seconds each. Total irrigation time was standardized at 1 minute and total irrigant volume, at 5 mL.

-NaOCl PUI+EA group - the canals were irrigated as described for the NaOCl PUI group, activated using an Irrisonic tip (Helse Ultrasonic), repeated 2 times for 20 seconds each, until 2 mm short of the working length. NaOCl was replaced with fresh irrigant and final agitation was performed with EA (Dentsply, Tulsa Dental Specialties), using the medium activator tip (#25/.04) to the working length for 20 seconds. Total irrigation time was standardized at 1 minute and total irrigant volume, at 5 mL.

-O_3_ group - the canals were irrigated with 5 mL of ozonated water at a concentration of 8 µg/mL for 1 minute, using a 0.55 x 20 mm needle (Embramac) inserted to a length of 10 mm.

-O_3_ PUI group - the canals were irrigated with 1.6 mL of ozonated water at a concentration of 8 µg/mL, using a 0.55 x 20 mm needle (Embramac) inserted to a length of 10 mm, activated using an Irrisonic tip (Helse Ultrasonic) coupled to an ultrasonic piezoelectric unit (J. Morita) at 30% power, inserted into the root canal until 2 mm short of the working length for 20 seconds and aspirated with a needle and syringe (Embramac). Ozonated water was replaced with fresh irrigant and agitation was repeated 3 times. Total irrigation time was standardized at 1 minute and total irrigant volume, at 5 mL.

-O_3_ PUI+EA group - the canals were irrigated as described for the O_3_ PUI group, activated using an Irrisonic tip (Helse Ultrasonic), repeated 2 times for 20 seconds each, until 2 mm short of the working length. Ozonated water was replaced with fresh irrigant and final agitation was performed with EA (Dentsply, Tulsa Dental Specialties), using the medium activator tip (#25/.04) to the working length for 20 seconds. Total irrigation time was standardized at 1 minute and total irrigant volume, at 5 mL.

Immediately after the disinfection procedures, the second microbiological sample (S2) was collected as described for S1, under aseptic conditions in a laminar flow hood under a 5% CO_2_ atmosphere, by inserting a sterile FM paper point (Tanari) to the working length for 1 minute and then placing it in an Eppendorf tube containing 900 µL of saline solution, which was vortex mixed for 30 seconds. The bacterial suspension was serially diluted at 10^0^, 10^-1^, and 10^-2^ concentrations, and aliquots were seeded into Petri dishes containing BHI agar and incubated at 37ºC under a 5% CO_2_ atmosphere for 24 hours. Microbial growth was measured by the CFU/mL counts. CFU counting was performed by a single calibrated and trained operator using a digital colony counter (Cp-600 Plus Tecnal).

S1 and S2 samples were kept frozen until DNA extraction for real-time qPCR analysis using the Maxima SYBR Green/ROX qPCR Master Mix detection system (Thermo Fisher Scientific, Waltham, MA, USA) in the 7500 Fast Real-Time PCR System (Applied Biosystems, Foster City, CA, USA).

The DNA of each previously isolated microorganism was extracted by using QIAamp DNA Mini Kit (Qiagen, Valencia, CA, USA) according to the manufacturer’s instructions. The DNA concentration (absorbance at 260 nm) was determined with a spectrophotometer (Nanodrop 2000; Thermo Scientific, Wilmington, DE, USA). After extraction, the sequences of the specific primers for the microorganisms studied were designed using the Primer-BLAST software from the National Center for Biotechnology Information and were as follows: *E. faecalis* (Forward: CCA ATC AAA TGG CGG CTT CTA CG, Reverse: GCG ATC AGG GAA ATG ATC GAT TCC); *C. albicans* (Forward: CGA TTC AGG GGA GGT AGT GAC, Reverse: GGT TCG CCA TAA ATG GCT ACC AG); and *S. aureus* (Forward: GCG ATT GAT GGT GAT ACG GTT, Reverse: AGC CAA GCC TTG ACG AAC TAA AGC). Prior to the real-time PCR assays, concentration-effect and melting curves were performed to determine the working concentration and specificity of the primers, respectively. The amplification reaction consisted of cycling at 50ºC for 2 minutes, at 95ºC for 10 minutes, followed by 40 cycles at 95ºC for 15 seconds and 60ºC for 1 minute. The results were analyzed based on the threshold cycle (Ct) value, which is the point corresponding to the number of cycles in which the amplification of the samples reached a threshold (determined between the fluorescence level of the negative controls and the exponential amplification phase of the samples) that allowed the quantitative analysis of the expression of the evaluated gene. The Ct values ​​were used to determine the absolute number of microorganisms in each experimental group, based on the previously established standard curve.

To calculate the percentage of microbial reduction per sample, the following formula was used, where MB is the total number of microorganisms before disinfection, obtained by collecting the first microbiological sample (S1), and MA is the total number of microorganisms after disinfection, obtained by collecting the second microbiological sample (S2):

$$\left(MB-100\%\right)*(\left(MB-MA\right)-X\%))$$

### Statistical analysis

The results were analyzed using Biostat 5.3 and tested for normality using the Shapiro-Wilk test. The data were not normally distributed and, therefore, analyzed using the nonparametric Kruskal-Wallis test, followed by Dunn’s test. The significance level was set at 5%.

## Results

When analyzing the cultures for the total count of viable microorganisms after root canal disinfection, there was a significant reduction in viable CFUs after using the following disinfection protocols: O_3_ PUI+EA, NaOCl, NaOCl PUI, and NaOCl PUI+EA (p˂0.05). Microbial counts before the various disinfection protocols did not differ significantly (p>0.05), indicating standardization of the study methodology. The most marked reduction in viable CFUs occurred in the NaOCl, NaOCl PUI, and NaOCl PUI+EA groups, with a significant difference in relation to the other groups. The combination of NaOCl with PUI+EA was the most effective in disinfecting the root canal system, reducing microbial counts to zero after the application of the protocol (p˂0.05) (Table 1). However, the percentage of microbial reduction did not differ significantly between the groups irrigated with NaOCl (Table 2).

**Table 1:** Microbial counts (log10 CFU/mL) in all experimental groups.

	Saline (control)	O₃	O₃ PUI	O₃ PUI + EA	NaOCl	NaOCl PUI	NaOCl PUI + EA	(p)
Before	0.39 (0.61)^Aa^	0.39 (0.68)^Aa^	0.64 (0.27)^Aa^	0.59 (0.35)^Aa^	0.36 (0.31)^Aa^	0.42 (0.52)^Aa^	0.44 (0.35)^Aa^	>0.05
After	0.64 (0.54)^Aa^	0.20 (0.40)^A1^	0.23 (0.15)^A1^	0.20 (0.16)^B1^	0.00 (0.30)^Bb^	0.00(0.15)^Bb^	0.00 (0.00)^Bb2^	<0.05
(p)	>0.05	>0.05	>0.05	<0.05	<0.05	<0.05	<0.05	

CFU: colony-forming unit; O₃: ozone; PUI: passive ultrasonic irrigation; EA: EndoActivator; NaOCl: sodium hypochlorite.

Data presented as median (interquartile deviation) and compared by the Kruskal-Wallis test, followed by Dunn’s test.

Different uppercase letters in the same column and different lowercase letters and numbers in the same row indicate statistically significant differences.

**Table 2:** Percentage of microbial reduction (CFU/mL) in all experimental groups.

	Saline (control)	O₃	O₃ PUI	O₃ PUI + EA	NaOCl	NaOCl PUI	NaOCl PUI + EA	(p)
%	3.08 (20.69)^A^	21.01 (35.9)^A^	51.39 (41.4)^A^	54.59 (33)^A^	100 (10.5)^B^	100 (9.2)^B^	100 (0)^B^	<0.05

CFU: colony-forming unit; O₃: ozone; PUI: passive ultrasonic irrigation; EA: EndoActivator; NaOCl: sodium hypochlorite.

Data presented as median (interquartile deviation) and compared by the Kruskal-Wallis test, followed by Dunn’s test.

Different uppercase letters in the same row indicate statistically significant differences.

When using the qPCR method for analysis, there was a significant reduction in the viable counts of *E. faecalis* and *C. albicans* after using the following disinfection protocols: O_3_ PUI, O_3_ PUI+EA, NaOCl, NaOCl PUI, and NaOCl PUI+EA (p˂0.05). Regarding *E. faecalis* and *C. albicans* counts after the various disinfection protocols, saline (control) and O_3_ were the least effective disinfecting agents, with a significant difference in relation to the other groups (p˂0.05) (Table 3).

**Table 3:** Microbial counts of *Enterococcus faecalis*, *Staphylococcus aureus*, and *Candida albicans* (qPCR) in all experimental groups.

	Saline (control)	O₃	O₃ PUI	O₃ PUI + EA	NaOCl	NaOCl PUI	NaOCl PUI + EA	(p)
*E. faecalis*								
Before	3.24 (1.96)Aa	5.37 (1.44)Ba	8.62 (6.92)Ba	8.36 (4.59)Ba	9.76 (12)Ba	9.05 (5.51)Ba	9.34 (8.38)Ba	<0.05
After	4.01 (0.82)Aa	2.02 (5.36)Aa	3.66 (2.18)Ab	1.61 (4.73)Ab	2.70 (1.39)Ab	1.97 (8.87)Ab	3.01 (2.68)Ab	>0.05
(p)	>0.05	>0.05	<0.05	<0.05	<0.05	<0.05	<0.05	
*S. aureus*								
Before	4.31 (3.27)Aa	3.81 (3.29)Aa	4.09 (4.29)Aa	2.97 (3.52)Aa	4.23 (6.07)Aa	2.19 (2.16)Aa	3.96 (2.24)Aa	>0.05
After	4.66 (2.46)Aa	1.24 (3.01)Ba	0.00 (0.00)Bb	0.00 (0.00)Bb	0.00 (0.00)Bb	0.00 (0.00)Bb	0.00 (0.00)Bb	<0.05
(p)	>0.05	>0.05	<0.05	<0.05	<0.05	<0.05	<0.05	
*C. albicans*								
Before	3.24 (2.6)Aa	7.56 (4.2)ABa	10.04 (8.3)Ba	9.83 (3.95)Ba	9.33 (5.25)Ba	9.31 (2.95)Ba	10.76 (9.50)Ba	<0.05
After	4.44 (2.07)Aa	3.68 (3.31)Aa	3.83 (5.55)Ab	2.65 (4.79)Ab	3.64 (5.73)Ab	3.37 (3.43)Ab	2.09 (3.64)Ab	>0.05
(p)	>0.05	>0.05	<0.05	<0.05	<0.05	<0.05	<0.05	

qPCR: quantitative polymerase chain reaction; O₃: ozone; PUI: passive ultrasonic irrigation; EA: EndoActivator; NaOCl: sodium hypochlorite.

Data presented as median (interquartile deviation) and compared by the Kruskal-Wallis test, followed by Dunn’s test.

Different uppercase letters in the same row and different lowercase letters in the same column indicate statistically significant differences.

**Table 4:** Percentage of microbial reduction of *Enterococcus faecalis*, *Staphylococcus aureus*, and *Candida albicans* (qPCR) in all experimental groups.

%	Saline (Control)	O₃	O₃ PUI	O₃ PUI + EA	NaOCl	NaOCl PUI	NaOCl PUI + EA	(p)
*E. faecalis*	0.00 (2.96)^A^	63.61 (28.78)^B^	64.36 (29.95)^B^	76.31 (33.65)^B^	65.39 (22.65)^B^	77.12 (24.83)^B^	77.69 (19.62)^B^	<0.05
*S. aureus*	0.00 (4.23)^A^	95.89 (63.77)^B^	100.00 (0.00)^B^	100.00 (0.00)^B^	100.00 (0.00)^B^	100.00 (0.00)^B^	100.00 (0.00)^B^	<0.05
*C. albicans*	0.00 (3.77)^A^	47.80 (37.33)^B^	65.80 (36.34)^B^	62.54 (47.75)^B^	61.92 (45.18)^B^	58.11 (29.45)^B^	70.66 (19.08)^B^	<0.05

qPCR: quantitative polymerase chain reaction; O₃: ozone; PUI: passive ultrasonic irrigation; EA: EndoActivator; NaOCl: sodium hypochlorite.

Data presented as median (interquartile deviation) and compared by the Kruskal-Wallis test, followed by Dunn’s test.

Different uppercase letters in the same row indicate statistically significant differences.

Regarding *S. aureus* counts after the various disinfection protocols, there was a significant microbial reduction in the O_3_ PUI, O_3_ PUI+EA, NaOCl, NaOCl PUI, and NaOCl PUI+EA groups (p˂0.05). When comparing groups regarding *S. aureus* counts, saline (control) was the least effective disinfecting agent, with a significant difference in relation to the other groups (p˂0.05) (Table 3).

When analyzing the percentage of microbial reduction, there was a significant reduction in the viable counts of *E. faecalis, S. aureus*, and *C. albicans* in all experimental groups in relation to the control group (p˂0.05), with no significant differences between the experimental groups (p>0.05) (Table 4).

## Discussion

According to the results obtained by the culture method, the first null hypothesis was rejected. The 8 µg/mL ozonated water and 2.5% NaOCl were not equivalent to each other, with 2.5% NaOCl being more effective. The second null hypothesis was also rejected because 8 µg/mL ozonated water combined with agitation improved the antimicrobial efficacy of this irrigant and 2.5% NaOCl combined with agitation yielded results superior to those of the other disinfection protocols. By the qPCR analysis, the first null hypothesis was rejected, since 8 µg/mL ozonated water without agitation performed worse than 2.5% NaOCl in the before-and-after comparison. The second null hypothesis was accepted for 2.5% NaOCl but rejected for 8 µg/mL ozonated water, as its effect was enhanced by agitation.

Several microorganisms and their byproducts are involved in the etiology of endodontic infections, including *E. faecalis,* which can invade dentinal tubules, shows strong adhesion to collagen, and is resistant to irrigating solutions commonly used during root canal instrumentation ^15^. Given the great ability of this microorganism to survive root canal disinfection, biofilms of *E. faecalis* have been widely used in research settings ^6^ ^,^ ^12^ ^,^ ^14^ ^,^ ^16^ ^,^ ^17^. However, because more than 150 species can colonize the root canal system ^2^, the present study used mature multispecies biofilms containing *E. faecalis*, *C. albicans*, and *S. aureus,* microorganisms that are equally resistant and contribute to persistent intracanal infection, as done in previous studies using multispecies biofilms ^15^ ^,^ ^18^.

In addition, it is important to highlight the biofilm formation time of 21 days, as used in previous studies ^12^ ^,^ ^15^ ^,^ ^17^ ^,^ ^18^. In contrast, there are studies with younger biofilms, such as the one conducted by Hubbezoglu et al. ^6^, who waited 24 hours for biofilm formation. Nogales et al. ^14^ used specimens inoculated with microorganisms that were incubated for 7 days, whereas Case et al. ^3^ waited 14 days for biofilm formation. According to Guerreiro-Tanomaru et al. ^19^, the susceptibility of the microorganism is related to the different growth phases, concluding that a 21-day biofilm is considered mature, as observed by confocal laser scanning microscopy. They also reported that the resistance mechanism of mature biofilms is complex and may involve changes in the penetration of the antimicrobial agent through the cell envelope, the production of enzymes that degrade antibiotics, and the increase in the exopolysaccharide matrix during biofilm development.

Despite being a potent antimicrobial agent, NaOCl in its different concentrations has disadvantages such as cytotoxicity, which encourages the investigation of other substances such as ozone, which has been used in the form of ozone gas, ozonated water, or ozonated oil as an optimal protocol for its use has yet to be established ^20^. The present study used ozonated water at a concentration of 8 µg/mL, in accordance with previous studies. Nogales et al. ^20^ investigated ozonated water at 2 µg/mL, 5 µg/mL, and 8 µg/mL concentrations in monospecies biofilms of *Pseudomonas aeroginosa*, *S. aureus*, and *E. faecalis* and concluded that the highest concentration was effective in eliminating the three bacteria. In a later study, in addition to investigating the antimicrobial efficacy of ozonated water at the three concentrations previously described, Nogales et al. ^14^ evaluated the cytotoxicity of ozone in human gingival fibroblasts and reported that the 8 µg/mL concentration was the most cytotoxic on first contact, with the recovery of cell viability over time, an important factor in cases of potential irrigant extrusion, such as in immature teeth, or even in situations of clear communication with the periodontium. One possibility is to hybridize these agents since ozonated water would possibly not be cytotoxic after using NaOCl, contrary to what occurs when NaOCl is mixed with chlorhexidine.

Agitation potentiates the effects of the irrigant, which can be agitated by PUI ^3^ ^,^ ^16^ ^,^ ^21^ ^,^ ^22^, continuous ultrasonic irrigation ^12^, sonic agitation using the EA system ^17^ ^,^ ^21^, and manual dynamic activation ^21^. In the current study, the irrigants were agitated by PUI, a technique with some limitations such as the Irrisonic tip working until 2 mm short of the working length. To overcome this limitation, we tested a hybrid technique by performing 2 cycles of PUI, followed by a final cycle of sonic agitation with an EA polymer tip to the working length. We are unaware of previous reports of hybridization of irrigant agitation, especially in clinical cases requiring endodontic treatment of curved canals. The advantage of a system such as EA (Dentsply, Tulsa Dental Specialties), or even Easy Clean (Easy Bassi, Belo Horizonte, MG, Brazil), would be the possibility of agitation of the irrigant along the entire working length, which constitutes a limitation of PUI.

The S1 and S2 samples were collected with sterile paper points, as previously described ^15^ ^,^ ^17^ ^,^ ^18^. However, a limitation of this method is that microorganisms can be collected only from the main canal and the most superficial dentin layer, in addition to not being able to identify disinfection by root canal thirds ^23^. Another sampling method involves stroking the canal walls with a Hedstroem file in order to collect bacteria from deep in the biofilm, as performed by Case et al. ^3^ and by Moraes et al. ^12^. However, according to Siqueira and Rôças ^23^, although files can obtain more visible material, this sampling method makes it difficult to control contamination, especially when the file is severed after sample collection, files cannot sample much deeper than paper points, and there are no studies demonstrating that one method is superior to the other. The authors also state that cryopulverization for sample preparation and DNA recovery is the best approach to overcome the limitations of the conventional paper point technique, but, because of its destructive nature, it should only be applied to root specimens obtained by endodontic microsurgery or extraction ^23^.

The current study used both culture and molecular (qPCR) methods to analyze the level of disinfection achieved with different irrigants and agitation methods, as was done in the study by Moraes et al. ^12^, differing only in that they used monospecies biofilms of *E. faecalis.* Although the culture method was used in many other studies ^3^ ^,^ ^6^ ^,^ ^15^ ^,^ ^17^ ^,^ ^18^, Siqueira and Rôças ^23^ report several limitations of the method, including the assessment of species prevalence only, without quantification, and the low sensitivity of culture for anaerobic species, which may lead to false-negative results. The real-time qPCR method overcomes these limitations by showing better sensitivity and specificity than the culture method and by producing quantitative results. In the current study, the qPCR assay was performed using SYBR Green, which is simpler and more accessible than TaqMan and has good sensitivity, although specificity is reduced because the dye binds to all double-stranded DNA present in the reaction, including nonviable microorganisms ^23^.

The results of our culture analysis showed that in the groups irrigated with 8 µg/mL ozonated water, the combination with PUI+EA agitation produced better results in the before-and-after disinfection comparisons, but the absence of this methodology in the literature precluded a direct comparison with the results of previous studies. Moraes et al. ^12^ assessed the antimicrobial efficacy of 8 µg/mL ozonated water with and without agitation using continuous ultrasonic irrigation and found no difference between the groups in the elimination of *E. faecalis.* In the study by Nunes et al. ^17^, activation of 40 ppm of ozonated water with the EA system enhanced disinfection, but the group treated with ozonated water with sonic agitation supplemented with photodynamic therapy had the best results.

In the present study, the results of culture analysis also showed that irrigation with 2.5% NaOCl had superior antimicrobial efficacy, with emphasis on the group with PUI agitation supplemented with EA, the only treatment regimen able to reduce microbial counts to zero. This result may have been caused by irrigation with NaOCl, well known as a potent disinfectant, supplemented with a hybrid agitation technique, thus obtaining the best of both techniques: cavitation and microacoustic streaming generated by PUI with the Irrisonic tip working until 2 mm short of the working length and sonic agitation to the working length. Previous studies comparing the antimicrobial efficacy of O_3_ and NaOCl obtained the same results ^8^ ^,^ ^12^ ^,^ ^17^, which suggest that NaOCl still seems to be the most reasonable irrigant option due to its ease of use, low cost, and no requirement for special equipment, unlike ozonated water that is stable only for a short period of time ^12^. According to Silva et al. ^8^, the success of ozone performance is strongly associated with the application protocol used, as ozone is dose-, time-, and bacterial strain-dependent. Our protocol included the application of 5 mL of 8 µg/mL ozonated water for 1 minute, which may explain the reduction in viable microorganisms, but not as effectively as in the specimens irrigated with NaOCl.

The qPCR results showed a significant difference in the reduction of *E. faecalis* counts in the groups irrigated with 8 µg/mL ozonated water, in the groups agitated by PUI and by PUI+EA, when comparing S1 and S2 samples. Similar results were reported by Hubbezoglu et al. ^6^, who obtained the same antimicrobial efficacy for ozonated water with PUI agitation and 5.25% NaOCl without agitation. These results were possibly achieved by the enhancement of ozonated water action with agitation. Moraes et al. ^12^ found no differences between the groups treated with ozone, both gaseous ozone and ozonated water, with and without continuous ultrasonic irrigation; 2.5% NaOCl was the most effective disinfecting agent. In their study, the irrigants were used in the same concentration and volume as in the present study, but for 3 minutes, and distobuccal roots of maxillary first molars were selected, which may explain the differences in the results ^12^.

*S. aureus* was the least resistant microorganism when comparing the disinfection protocols vs control (saline). Therefore, 8 µg/mL ozonated water and 2.5% NaOCl, both with and without agitation, significantly reduced *S. aureus* counts. Comparing different concentrations of ozonated water without agitation, Nogales et al. ^20^ also reported a reduction in the viable counts of *S. aureus*. Nogales et al. ^14^ also found similar results when comparing the antimicrobial efficacy of 1% NaOCl with gaseous ozone and ozonated water as a complementary therapy, as did Pinheiro et al. ^18^, who compared root canal disinfection efficiency of instrumentation associated with irrigation with 2.5% NaOCl, 2% chlorhexidine, and 40 µg/mL ozonated water and also reported a reduction in the biofilm of *S. aureus*. According to Estrela et al. ^24^, this result may be explained by the fact that, when present in mixed infections, *E. faecalis* is the dominant microorganism.

*C. albicans* was found to be significantly reduced by irrigation with 8 µg/mL ozonated water with agitation and by irrigation with 2.5% NaOCl with and without agitation in our before-and-after comparisons of disinfection protocols. However, none of the treatment regimens were able to reduce microbial counts to zero. Huth et al. ^25^ reported complete eradication of *C. albicans* only when 5.25% NaOCl was used, followed by a reduction of more than 96% with gaseous ozone, ozonated water, and chlorhexidine. Cardoso et al. ^15^ examined the effects of ozonated water on biofilms of *C. albicans* and *E. faecalis* and reported a reduction in CFUs for both microorganisms. Both studies, however, used only the culture method for analysis.

It is important to note that there was a reduction in the total count of viable microorganisms in all experimental groups, an extremely satisfactory outcome according to Siqueira and Rôças ^1^. Complete eradication of the microorganisms was achieved by NaOCl PUI+EA in all specimens in the group when analyzed by the culture method, which did not occur when disinfection efficiency was analyzed by the qPCR method. *S. aureus* was eliminated by irrigation with ozonated water with agitation and by irrigation with NaOCl with and without agitation, but the other microorganisms under study were present in all groups, supporting that the usefulness of the culture method is limited.

Despite the positive aspects, such as studying multispecies biofilm, researching the hybridization of irrigant agitation, in an attempt to overcome the limitations of sonic and ultrasonic agitation methods, and studying two different methodologies to quantify the disinfection of samples, this study has limitations inherent *in vitro* research, such as the difficulty in reproducing the biofilm as it is organized *in vivo*.

It can be concluded that there was a reduction in microorganisms in all experimental groups. By the culture method, 2.5% NaOCl agitated in a hybrid way sterilized all specimens, whereas by the qPCR method, 2.5% NaOCl with and without agitation, as well as 8 µg/mL ozonated water with its action enhanced by the agitation techniques, was effective in reducing mature multispecies biofilms containing *E. faecalis*, *C. albicans*, and *S. aureus*. Also, the antimicrobial efficacy of the irrigant is related to the microorganism present in the root canal system.
